# Development of CRISPR-Mediated Nucleic Acid Detection Technologies and Their Applications in the Livestock Industry

**DOI:** 10.3390/genes13112007

**Published:** 2022-11-02

**Authors:** Xuying Zhang

**Affiliations:** Institute for Animal Breeding and Genetics, University of Veterinary Medicine Hannover, Bünteweg 17p, 30559 Hannover, Germany; xuying.zhang@tiho-hannover.de

**Keywords:** Cas12, Cas13, Cas9, Cas14, CRISPR-associated proteins, livestock, nucleic acid detection, infectious diseases, meat/milk component, sex determination

## Abstract

The rapid rate of virus transmission and pathogen mutation and evolution highlight the necessity for innovative approaches to the diagnosis and prevention of infectious diseases. Traditional technologies for pathogen detection, mostly PCR-based, involve costly/advanced equipment and skilled personnel and are therefore not feasible in resource-limited areas. Over the years, many promising methods based on clustered regularly interspaced short palindromic repeats and the associated protein systems (CRISPR/Cas), i.e., orthologues of Cas9, Cas12, Cas13 and Cas14, have been reported for nucleic acid detection. CRISPR/Cas effectors can provide one-tube reaction systems, amplification-free strategies, simultaneous multiplex pathogen detection, visual colorimetric detection, and quantitative identification as alternatives to quantitative PCR (qPCR). This review summarizes the current development of CRISPR/Cas-mediated molecular diagnostics, as well as their design software and readout methods, highlighting technical improvements for integrating CRISPR/Cas technologies into on-site applications. It further highlights recent applications of CRISPR/Cas-based nucleic acid detection in livestock industry, including emerging infectious diseases, authenticity and composition of meat/milk products, as well as sex determination of early embryos.

## 1. Introduction

As in humans, large-scale recurring epidemics dramatically affect livestock populations, especially food-producing animals such as pigs and cattle. Infectious diseases caused by pathogens such as African swine fever virus (ASFV, family *Asfarviridae*) [1], porcine reproductive and respiratory syndrome virus (PRRSV, family *Arteriviridae*) [2], porcine epidemic diarrhea virus (PEDV, family *Coronaviridae*) [3], encephalomyocarditis virus (EMCV, family *Picornaviridae*) [4] and classical swine fever virus (CSFV, family *Flaviviridae*) [5] have induced reproductive failures, high mortality and trade restrictions, bringing serious commercial damages to the swine industry [6]. Bovine viral diarrhea virus (BVDV, family *Flaviviridae*) and lumpy skin disease virus (LSDV, a member of the genus *Capripoxvirus* and family *Poxviridae*), etc., are economically important infectious agents of cattle worldwide. In addition, pathogenic bacteria and parasites affect animal production and animal welfare, causing significant economic losses to the livestock industry [7,8,9,10]. Livestock production in large communities promote spreading and maintaining huge pathogen populations and facilitate mutation and evolution of pathogens [11,12,13], which further complicates the prevention and control of infectious diseases. Several molecular diagnostic approaches have been developed and applied for identifying and quantifying a wide range of pathogens. Reverse transcription polymerase chain reaction (RT-PCR), reverse transcription quantitative polymerase chain reaction (RT-qPCR) and droplet digital PCR (ddPCR) are effective methods of pathogen detection [14,15,16]; however, these are not suitable for use in the field due to their dependence on sophisticated/expensive equipment, long testing time and highly skilled personnel. Isothermal diagnostic approaches, such as reverse transcription loop-mediated isothermal amplification (RT-LAMP) and recombinase polymerase amplification (RPA) [17,18,19], have the following advantages: amplification at single temperature eliminates the need of bulky, advanced and expensive equipment, making them cost effective and potential for detection at the point of care (POC); compared to RT-PCR (2–4 h), the turnaround time (~1 h) is less; these methods enable naked eye visualization as well as real-time monitoring [19,20]. Nevertheless, detailed analyses of these isothermal amplification methods indicate their low sensitivity, low specificity, and low throughput [21,22,23]. The multiplexed primer pairs in LAMP may yield false-positive assay results [24,25]. LAMP is less sensitive to complex samples such as blood and tissues due to possible inhibitors of Bst polymerase [26]. To effectively prevent and control infectious diseases, it is desirable to establish a feasible, sensitive, specific, and reliable on-site diagnostic strategy for nucleic acid detection.

First described in bacterial genomes 30 years ago [27], the clustered regularly interspaced short palindromic repeats and the associated protein (CRISPR/Cas) system was reported in 2005 to operate as a natural defense mechanism of bacteria and archaea against viral and plasmid DNA infections [28]. CRISPR/Cas systems can be categorized into Class 1/Class 2. The Class 1 systems, including type I, type III and type IV, are characterized by multi-subunit-protein complexes. The Class 2 systems (type II and type V and type VI) consist of a single effector protein (known as “Cas”), which is large, polydomain and polyfunctional, as well as a guide CRISPR RNA (crRNA). CRISPR/Cas Type II is distinguished by the existence of Cas9 and a chimeric single guide RNA (sgRNA). The ribonucleoprotein (sgRNA + Cas9) identifies a protospacer adjacent motif (PAM) positioned at the G-rich 3′ end of a double-stranded DNA (dsDNA) target, which activates Cas9 nuclease for the induction of a blunt-end double-strand break (DSB) on the target DNA [29]. CRISPR/Cas type V systems are primarily characterized by a set of effector proteins Cas12 (Cas12a/Cpf1, Cas12b/C2c1, etc.) and crRNA, which increases the multiplexing capacity of the type V. The crRNA + Cas12 complex recognizes a T-rich 5′-end-localized PAM (not G-rich PAM) and generates staggered 5′-end dsDNA breaks (cis-cleavage activity) [30]. The target binding of crRNA + Cas12 also induces non-specific trans-cleavage of single-stranded DNA (ssDNA) [31]. CRISPR/Cas type VI systems (A, B, C and D subtypes) have Cas13 as the effector protein. Upon recognition of a single-stranded RNA (ssRNA) target, crRNA + Cas13 complex induces a blunt-end break (cis-cleavage activity) and indiscriminate degradation of any neighboring ssRNA (trans-cleavage activity) [32]. Since the first CRISPR/Cas-based diagnostic tool was reported in 2016, a large number of CRISPR Class 2-based diagnostic systems have been emerging, especially during the COVID-19 pandemic in 2020–2022 (Table 1). Likewise, since 2020, the “collateral” cleavage properties of CRISPR/Cas12 and CRISPR/Cas13 have been further widely used for in vitro detection of different pathogens, including viruses, bacteria and parasites in livestock (Table 2).

This review summarizes research efforts to improve the sensitivity, speed, affordability and field deployability of CRISPR/Cas-mediated diagnostics occurring worldwide. It also provides a summary of using CRISPR/Cas diagnostics to detect/diagnose/genotype various (non)pathogenic nucleic acids in livestock.

## 2. Development of CRISPR/Cas-Based Nucleic Acid Detection Systems

### 2.1. Cas Type II Based Diagnostic Platforms

**Figure 1:**
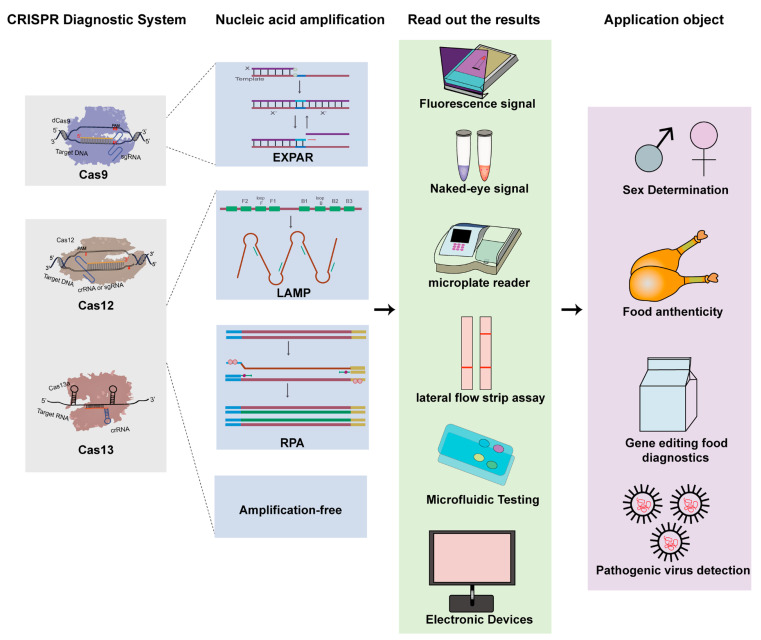
Schematic principle of clustered regularly interspaced short palindromic repeats (CRISPR) Class 2 systems in nucleic acid detection and the applications of CRISPR and the associated protein systems (CRISPR/Cas)-based diagnostics in livestock industry.

Most of the early CRISPR-based diagnostic inventions, from 2016 to 2019, relied on the Cas9/type II systems (Table 1, Figure 1), which by themselves were not able to elicit a strong/specific signal when target nucleic acids exist in the sample. These technologies employ different design strategies based on sgRNA/Cas9 and require pre-amplification (paired dCas9 (PC) reporter system [35]) or post-amplification (CRISPR-typing PCR (ctPCR) [37,40], CRISPR- or Cas9/sgRNAs-associated reverse PCR (CARP) [39], finding low abundance sequences by hybridization-next generation sequencing (FLASH-NGS) [41]) for target nucleic acids by PCR. These approaches compromise the vast promise of CRISPR diagnostics. Since the complex, specific and detailed steps involved in the methods make single-tube detection almost impossible, resulting in a possible chance of contamination during the diagnosis. Although PCR is the most famous amplification technique, the requirement for thermal cycling limits the non-laboratory applications of these CRISPR/Cas9-based methods.

Additional CRISPR/Cas9-mediated diagnostics such as nucleic acid sequence based amplification-CRISPR cleavage (NASBACC) [33], CRISPR/Cas9-triggered nicking endonuclease-mediated strand displacement amplification method (CRISDA) [38], and CRISPR/Cas9 triggered isothermal exponential amplification reaction (CAS-EXPAR) [36] involved isothermal amplification, avoiding the need for thermal cycler in conventional PCR and thus taking a major step towards POC diagnostics. NASBACC can distinguish viral strains with single-base resolution, relying on isothermal RNA amplification combined with toehold switch sensors [33]. Unlike traditional amplification reactions, such as in CRISDA, CAS-EXPAR does not need any exogenous primers and thus has been shown to be more specific for mutant targets [36]. The pioneering success story of CRISPR/Cas9-mediated diagnostics has inspired other CRISPR/Cas systems, such as lateral flow-based and fluorometer-based diagnostics [106,107].

### 2.2. Cas Type V and VI Based Diagnostic Platforms

Preliminarily in 2016, C2c2 (now known as Cas13a) was observed to catalyze ssRNA cleavage in presence of single crRNA and complementary protospacer [32]. Furthermore, in 2017, the Gootenberg group confirmed that Cas13a (previously known as C2c2) exhibits RNA targeting collateral activity which can be conducted at isothermal conditions and based on Cas13a developed a novel system named specific high-sensitivity enzymatic reporter unlocking (SHERLOCK), used for point-care diagnostics [65]. It can detect target RNA or DNA (in vitro transcription required), with attomolar sensitivity and specificity of single-base mispairs [65]. One year later, the second version (SHERLOCKv2) was reported with advances: quad-channel monoreactive multiplexing with orthogonal CRISPR/Cas; quantitative measuring for ~2 attomoles inputs; 3.5-fold improvement of sensitivity by combining Cas13 with Csm6; and lateral flow readout without any additional device [66].

In 2018, Li et al. developed the one-hour low-cost multipurpose highly efficient system (HOLMES) using the incidental cleavage activity of Cas12a on non-target ssDNA and a fluorescent reporter linked to ssDNA [43]. The authors demonstrated that when combined with PCR, HOLMES achieved SHERLOCK sensitivity (attomolar level), better than PCR or qPCR alone [43]. HOLMES was validated for the detection of DNA and RNA (requiring reverse transcription into cDNA) viruses [43]. Its version 2 (HOLMESv2) replaces Cas12a with Cas12b, enabling not only molecular diagnostics but also epigenetic applications, such as quantification of DNA methylation level [31,47]. The combination of Cas12a ssDNase activation with isothermal amplification was validated in 2018 and named as DNA endonuclease-targeted CRISPR trans reporter (DETECTR), also with attomolar sensitivity [31]. In the same year, a diverse CRISPR family containing Cas14 was identified to be similar to type V [64]. Unlike Cas12 with low fidelity in discriminating against ssDNA substrates, Cas14 requires complementarity in the seed region to recognize ssDNA substrates, which property raises its possibility to detect SNPs without the constraint of a PAM sequence [64].

One-tube reaction system: In spite of the impressiveness and rapid development of CRISPR/Cas-based detectors, shift of reactants to another tube and the reliance on nucleic acid isolation may result in a high chance of contamination during the test process and false positives. One-tube detection platform by RT-RPA and CRISPR/Cas12 (DETECTR) was deployed for COVID-19 detection [55]. The platform achieved single-tube testing by physically separating the two reaction components during amplification and mixing them by centrifugation afterwards [55]. A single-tube assay platform with RPA and CRISPR/Cas13a (SHERLOCK) also works [55]. A primary comparison of DETECTR and SHERLOCK indicated that their testing effectiveness was essentially similar, despite the more complex components of SHERLOCK [55]. Nucleic acid extraction, however, was still required prior to these single-tube assays.

Heating unextracted diagnostic samples to bliterate nucleases (HUDSON) is a methodology using heat and chemical reduction to lyse virus particle and deactivate high-level RNases present in body fluids [67]. Combining HUDSON with SHERLOCK, field-deployable diagnostic platforms were developed for the detection of viruses from body fluids without the need for a nucleic acid extraction step [67,69]. The in-tube fluorescence readout method also reduced the risk of contamination as the reaction tubes remained closed [69]. SHERLOCK testing in one pot (STOP) platform also combines LAMP and CRISPR/Cas12b-based detection in a single tube, but requires an extraction step using magnetic beads prior to the testing, which raises the cost and handling time, and is still unable to improve the sensitivity (200 copies/reaction) to the level of RT-qPCR (20 copies/reaction) [50]. Instead of using canonical PAM, a suboptimal PAM-mediated method (sPAMC) reported in 2022 appears to be the first real one-tube detection methodology without the need for RNA extraction [62]. The decreased binding affinity of CRISPR/Cas12a to the suboptimal PAM substrate diminished its cis-cleavage activity, facilitating the shift of equilibrium to isothermal amplification and thereby leading to stronger fluorescence. The test time is within 20 min and the sensitivity is similar that of RT-qPCR [62].

Amplification-free strategies: Some novel amplification-free detection strategies, based on CRISPR/Cas12a or CRISPR/Cas13a, were reported as potentially more suitable POC sensors for viral nucleic acids. In 2019, an electrochemical biosensor (E-CRISPR) was reported based on the trans-cleavage activity of Cas12a and electrode consisting non-specific ssDNA, to convert target recognizing into electrochemical signal. Not only for nucleic acid sensing, the system can also be utilized for protein sensing [45]. Another E-CRISPR was developed in 2020 with immobilized low surface coverage and morphologically uncompact hpDNA which supplies approachable substrates for effective Cas12a cleavage, resulting in higher sensitivity than that of conventional ssDNA [53]. Additionally in 2021, an amplification-free fluorescent biosensor was created via a metal-enhanced fluorescence (MEF) with DNA-functionalized Au nanoparticles (AuNP). MEF color changes from purple to red-purple when target DNA-activated CRISPR/Cas12a degrades ssDNA between AuNP and fluorophore [57]. Enhanced analysis of nucleic acids with CrRNA expansions (CRISPR-ENHANCE), another CRISPR/Cas12a-based detection system, employs genetically engineered crRNA with specific 7-mer’-expansions and optimized working conditions. Without the need for target pre-amplification, it achieves femtomolar level sensitivity [59].

In addition, Fozouni et al. developed a CRISPR/Cas13a-multiple crRNAs assay for rapid, POC detection, which improved sensitivity not by target amplification, but by activating more Cas13a per target RNA, enabling direct conversion of the fluorescent signal to viral load [71]. Ultralocalized Cas13a detection confines the RNA-activated CRISPR/Cas13a system to cell-sized reactors by droplet microfluidics to simultaneously increase target and reporter local concentrations. By comparison with the bulk Cas13a assay, it realizes a >10,000-fold sensitivity improvement and achieves absolute digital single-molecule RNA quantification [72]. In 2022, a dual enzyme amplification scheme that combines target-induced Cas activation with a following release of the enzymatic reporter—horseradish peroxidase (HRP)—into solution, has achieved quick, convenient (25 °C) and sensitive (~10 fM) detection of nucleic acids, without the need for PCR [74]. CRISPR/Cas13a-graphene field-effect transistors (gFETs) method, may be one of the most sensitive amplification-free diagnostic systems so far and has been validated for the detecting of SARS-CoV-2 and respiratory syncytial virus as low as 1 aM [73].

Multiplexed pathogen detection system: The combinatorial arrayed reactions for multiplexed evaluation of nucleic acids (CARMEN) platform was reported in 2020 for scalable multiplexed pathogen detection. Nanoliter droplets of CRISPR/Cas13/crRNA reaction reagents are self-organized in a micropole array, paired with amplified sample droplets, to detect each sample repeatedly for each crRNA [68]. CARMEN system combined with Cas13 has been reported to effectively screen over 4500 crRNA candidates against desired targets through a single array platform. CARMEN can be easily scaled for practical use due to its inherent multiplexing, throughput capabilities, as well as 300 times reduction in reagent cost per test due to miniaturization [68]. The authors demonstrated that CARMEN-Cas13 enables the simultaneous detection of all 169 human-related viruses, as well as subtypes of influenza A strains and HIV drug resistance variations [68]. The CARMEN panel has also been applied to simultaneously detect up to 21 respiratory viruses [108], 52 clinically relevant bacterial species as well as a number of key antibiotic resistance genes [109], demonstrating its diagnostic-grade performance in both academic and clinical settings [108].

Rapid Visual CRISPR Assays: In 2022, Xie et al. systematically screened and identified nine ssDNA-FQ reporters suitable for CRISPR/Cas12a-based visual colorimetric detection, with particularly strong performance of ROX-labeled reporter [61]. A convolutional neural network algorithm was also developed and implemented in the MagicEye mobile app to enable standardization and automation of image analytical colorimetric evaluation [61]. By Combining the DETECTR strategy [31] and the ROX-labeled reporter, RApid VIsual CRISPR (RAVI-CRISPR) has been established as a device-less (only a portable rechargeable incubator is required) single-tube colorimetric POC platform [61]. The RAVI-CRISPR has been successfully applied for detection of SARS-CoV-2 [61], ASFV [61], and JEV [86], for sex determination in pigs [101], as well as for recognition of meat species and meat products [104]. The RAVI-CRISPR in these applications has a LOD of 2–9 total copies and takes only 35–60 min using naked-eye colorimetric detection. The RAVI-CRISPR/MagicEye system appears to be a breakthrough technology for rapid pen- or bed-side detection.

### 2.3. Software for Designing CRISPR/Cas-Based Nucleic Acid Assays

An increasing number of novel RNA-guided CRISPR endonucleases, such as Cas9 from various types of bacteria, Cpf1 nuclease, C2c1, C2c2, and C2c3 systems, have been discovered with a different PAM. The guide RNA sequence directly affects the target-induced cleavage efficiency and non-intentional off-target binding and cleavage. Therefore, the design of efficient and specific guide RNA is crucial for the successful application of CRISPR/Cas-mediated diagnostics. A list of CRISPR design tools have been created and some popular guide RNA design tools, such as CRISPOR [110], CHOPCHOP [111], Off-Spotter [112], Cas-OFFinder [113], CRISPR-Era [114], and E-CRISP [115], are available with GUIs for ease of use. Some of these tools, such as Off-Spotter [112] and Cas-OFFinder [113], were developed specifically for detecting potential off-target editing. Other tools, such as CHOPCHOP [111] and CRISPR-OFFinder [116], are not only for Cas9 but also provide options for alternative Cas nucleases and PAM recognition. CRISPR-OFFinder is a versatile tool to rapidly design sgRNAs for different CRISPR/Cas systems with minimal off-target effects, particularly for Cpf1 and C2c1 [116]. User-defined PAM and sgRNA length are supported to enhance targeting specificity [116]. While CRISPR-RT [117] and CRISPR-DT [118] were developed to help scientists design gRNAs for the CRISPR/Cas13a/C2c2 and CRISPR/Cas12a/Cpf1 systems, respectively. To quickly discover or score hundreds of CRISPR targets, command-line tools like FlashFry [119] are also available. They are fast and flexible with the output presented in easy-to-operate text files.

### 2.4. Readout Methods

Many CRISPR/Cas-based diagnostics have been developed with different readout methods, and major attempts have been made to achieve POC testing. The fluorescence and lateral flow assay are so far the most used readout methods in CRISPR/Cas-based detection platforms. Other signal detection methods have also been reported, such as using electrochemical biosensors (E-CRISPR [45,95], PGMs-CRISPR [54], MOECS [120]), chemiluminescence enhancement biosensors (CRICED [121], CLE-CRISPR [122]), toehold switch-linked colorimetry (NASBACC [33]), and potentiometry (CRISPR-Chip [42]). Some CRISPR/Cas-based diagnostic technologies allow for fluorescent signals to be read by the naked eye under blue light (CRISPR-Cas12a-NER [48], opvCRISPR [56], CASdetec [49]) or to be measured with a mobile phone (multiple enhanced CRISPR-Cas13 assay [71], SHINE [69]). Naked-eye colorimetric detection (RAVI-CRISPR [61], CRISPR-Cas13a/HRP assay [74]) reported in 2022 is probably simplest-to-date reading method for molecular POC testing, without fluorescence detector or mobile phone required for visualization. 

## 3. Current Applications of CRISPR/Cas-Based Nucleic Acid Detection Technologies in Livestock

### 3.1. CRISPR/Cas-Based Detection of Pathogenic Viruses in Livestock

ASFV is a nucleocytoplasmic large DNA virus that is highly infectious and pathogenic [123]. To date, there is no available vaccine or antiviral drug against ASFV, but CRISPR-based methods of ASFV detection to control ASF transmission. Lin et al. established CRISPR/Cas9 eraser-based PAM-implanted PCR combined with lateral flow endpoint detection method [75] (Table 2, Figure 1). Pretreatment of PCR mixture with Cas9/sgRNA to selective clean up contamination amplicons abolishes false-positive amplification. However, if the source of contamination is unknown, sequencing must be employed prior to designing new primers and sgRNAs [75]. The CRISPR/Cas9 erase method requires costly instrumentation and specialized handling system, therefore remains unsuitable for fast clinical testing [75]. Reported AssSFV diagnostic assays combing the incidental cleavage activity of Cas13a (CRISPR/Cas13a-LFD [78]) or Cas12a (RAVI-CRISPR [61,76]) with isothermal amplification solved this problem. A water bath/portable rechargeable hand warmer and a pipette are the main devices required to perform these assays, indicating their potential in-field applicability for ASFV detection. These methods have high sensitivity (LoD: 7–10 copies/µL) and high specificity [61,76,78]. The single-tube reaction in these methods reduces the likelihood of contamination. Compared to CRISPR/Cas13a-LFD [78], RAVI-CRISPR [61,76] is even more advanced and cheaper, as it does not require lateral flow strip or fluorescence detector. RAVI-CRISPR achieves accurate colorimetric naked-eye detection using a ROX-labeled reporter [61,76]. The CRISPR/Cas12a/multiplex-crRNA system was designed for direct detection of ASFV DNA without nucleic-acid preamplification [79]. Its detection limit (~1 pM) is 6–64 times stronger than that of the conventional single-crRNA CRISPR/Cas12a system [79]. It also reduces the possibility of detecting losses due to naturally occurring mutations in viral genes [79]. In the future, the CRISPR/Cas12a/multiplex-crRNA method could be further combined with naked-eye colorimetric detection using a ROX-labeled reporter [61,76], making the diagnostic platform more deployable in the field. In reality, ASFV, CSFV, and APPV are co-endemic in many areas, causing highly similar clinical symptoms. So far, a multiplex RT-PCR assay [124] is available to test these viruses simultaneously, but it requires specialized instruments and skilled personnel. The multiplex isothermal amplification in combination with CRISPR/Cas12a assay, which has successfully distinguished PEDV, TGEV, PDCoV, and SADS-CoV [83] would have the potential to simultaneously and differentially detect these three viruses in the field.

PRRSV is a positive-sense RNA virus, and can cause abortion of pregnant sows, and respiratory symptoms and death in pigs [125]. Visual nucleic acid detection methods based on CRISPR/Cas13a [25] or CRISPR/Cas12a [80], respectively, have been established, since PRRSV detection approaches based on antigen-antibody response [126], PCR or RT-qPCR [127] are not applicable for poorly equipped laboratories or on-site diagnostics with high sensitivity. The sensitivity of the CRISPR/Cas13a-based method for PRRSV detection is 172 copies/reaction, similar to that of RT-qPCR [128,129], while the CRISPR/Cas12a assay is much more sensitive and can reach the sensitivity of one copy/reaction within 25 min [80]. Both CRISPR/Cas-based assays have been successfully deployed in clinical samples from diverse farms [25,80].

PEDV, an enveloped, positive RNA virus, induces acute intestinal infections manifested by severe dehydration, diarrhea, nausea and high rate of mortality in piglets [130,131]. PEDV can be classified into genotypes GI and GII according to mutations in spike (*S*) gene [132]. The CV777 vaccine strain has been created based on PEDV GI genotype and widely used to control GI PEDV infection in pigs [133]. Whereas, the GII genotype is commonly reported in cases of immune failure and has become a main prevalent PEDV strain [134,135]. For distinguishing PEDV-attenuated vaccine strains and wild-type virus strains, an RT-ERA-CRISPR/Cas12a assay was developed with high sensitivity (LOD of two copies) based on a 51nt deletion mutation in the open reading frame 3 (*ORF3*) gene [81]. To decide if a pig should be immunized with the CV777 vaccine, an RT-RAA-CRISPR/Cas12a platform targeting the *S* gene was created for the detection of GII PEDV [82]. Additionally, Liu et al. (2022) developed a single-tube multiplex RT-LAMP-Cas12a diagnostics to simultaneously detect TGEV, PDCoV, SADS-CoV, and PEDV, although it cannot recognize different PEDV strains [83]. With naked-eye colorimetric detection, it has a LOD of one copy and takes only 25 min [83]. These CRISPR/Cas12a-based assays, with different detection purposes, have promising potential for the prevent and control of PEDV worldwide.

Besides, the strategy of combining isothermal amplification with the collateral cleavage activity of CRISPR/Cas12a (DETECTR [31]) or CRISPR/Cas13a (SHERLOCK [65]) has also been applied for the detection of PCV3 (ssDNA circovirus, ERA-CRISPR/Cas12a) [84], PPV (non-enveloped DNA virus, ERA-CRISPR/Cas12a) [85], EMCV (non-enveloped ssRNA virus, RAA-CRISPR/Cas13a) [87], JEV (ssRNA virus, RT-LAMP-CRISPR/Cas12a) [86], LSDV (linear dsDNA virus, RPA-Cas12a) [88], BVDV (positive-sense ssRNA virus, CRISPR-Cas13a-based) [89], and CaPV (LAMP-CRISPR/Cpf1) [90]. These diagnostic methods have robustness, convenience, sensitivity, specificity, affordability and potential adaptation for in-field detection or surveillance of the viruses in clinical and vector samples.

### 3.2. CRISPR-Based Detection of Pathogenic Bacteria and Parasites in Livestock

Compared to Cas13a-based SHERLOCK [65], Cas12a-based DETECTR [31] may be a more desirable strategy for the detection of bacterial nucleic acids without the need for in vitro transcription, since Cas12a is a DNA endonuclease. *Brucella spp.* can cause widespread brucellosis in cattle, sheep, pigs, as well as dogs [136]. Four humankind pathogens of Brucella spp. (*Brucella abortus*, *Brucella melitensis*, *Brucella canis*, and *Brucella suis*) can transmit between humans and animals, leading to occupational risks for livestock workers [137,138]. The development of an RPA-CRISPR/Cas12a diagnostics with both fluorescent and electrochemical signal readout methods enabled rapid and accurate detection of these four Brucella strains in blood and milk samples [95]. The dual-signal readout approach improves the accuracy of the assay, and the authors demonstrate that this method has better diagnostic performance than real-time PCR [95]. Additionally, the RPA-CRISPR/Cas12a strategy has been used for the detection of other foodborne pathogenic bacteria (*Listeria monocytogenes*, *Yersinia enterocolitica*, *Staphylococcus aureus*, *Campylobacter jejuni*, *Escherichia coli*, etc.) in food production animals at attomolar level [96,97,98,99,100].

Traditional parasite diagnostic approaches, such as light microscopy and immunoassay, are not reliable and require a large quantity of samples [139]. *Cryptosporidium parvum* is a zoonotic important intestinal protozoan parasite and can induce cryptosporidiosis in humans and domestic/wild animals all over the world [10]. A diagnostic method based on RPA-Cas12a/crRNA (ReCTC) has been validated for the detection of *C. parvum* subtype family IId from clinical human and bovine fecal samples [94]. In future studies, the ReCTC assay could be further optimized as a one-tube reaction for faster and simpler on-site diagnosis. *Toxoplasma gondii* is a globally distributed protozoan parasite, causing life-threatening consequences in immunocompromised patients [140] as well as abortions and stillbirths in livestock [141,142]. In combination with RPA or RAA, CRISPR-Cas12a/Cas13a based methods have been reported for quick in-field detection of *T. gondii* [91,92,93]. The authors demonstrated that the RAA-Cas12a/Cas13a systems have advantages over conventional PCR-based method in terms of conveniency and sensitivity [92,93]. In terms of reducing cross-contamination and cost, the RPA-CRISPR/Cas12a assay (single-tube strategy and 100 nM of Cas12a) is more advanced than the RAA-CRISPR/Cas12a assay (two separate processes and 800 nM of Cas12a) [91]. Applications of these novel methods potentially contribute to the control of toxoplasmosis in humans and animals.

### 3.3. CRISPR Assays for Sex Determination and Meat/Milk Products

Sex determination of early embryos is a requisite for the achievement of sex control and ideal male/female ratios [143]. It has a huge impact on breeding efficiency and worldwide animal production, such as milk yield and weight gain [143,144,145]. Previous reports have also indicated that pork quality depends on sex-related physiology [146,147] and sex-expressed genes [148]. A RAVI-CRISPR/LAMP-CRISPR-Cas12a system targeting zinc finger protein X-linked (*ZFX*) and sex-determining region Y (*SRY*) genes was established for sex identification of early pig embryos and pork with simplicity, cheapness, sensitivity, and specificity [101]. Compared to the traditional methods such as PCR and fluorescence-activated cell sorting (FACS), it can be easily performed in the field and does not require technical expertise. Its fluorescence signal can be checked with the naked eye, a portable UV/blue transilluminator/luminescent flashlight [101]. The RAVI-CRISPR strategy also has potential in determining the sex of other livestock species.

Meat and meat products are an indispensable part of human diet and meat/food safety is a high concern worldwide. Meat adulteration causes serious economic and health consequences globally and harms the religious beliefs of Muslim consumers [149]. Conventional methods for the identification of animal/meat products, such as enzyme-linked immunosorbent assays (ELISA) [150] and chromatographic methods [151], demand knowledgeable personnel and are expensive, inaccurate, and time-consuming. Molecular techniques, particularly CRISPR/Cas12-based methods targeting species-specific DNA have been reported for meat identification. The CRISPR/Cas based PCR DNA barcoding method (CAPCOD), integrating CRISPR/Cas12 system and PCR, can identify 0.1% (*w*/*w*) pork contamination in raw meat mixtures [102]. It has been used for identifying pork content in complex processed (non-)halal foods [102]. This method is fast and specific, but requires complex instrumentation for PCR, limiting its application in-field detection. The DETECTR strategy combining isothermal amplification (instead of PCR) and the CRISPR/Cas12a detection technique [31] can overcome this problem. An RPA-CRISPR/Cas12a method has been developed for pork detection and validated on beef and pork mixtures under raw, cooked and high-pressure conditions [103]. As low as 10^−3^ ng of porcine genomic DNA can be identified by the portable box in 30 min [103]. The authors confirmed that the results were consistent with the real-time PCR method. The RPA-CRISPR/Cas12a method can therefore be used for in situ pork detection with high speed, accuracy and sensitivity [103]. In addition, LAMP-CRISPR/Cas12a with a Texas red-labeled ssDNA reporter for visual colorimetric detection (RAVI-CRISPR) has been reported to detect meat species of pig, chicken and duck with high sensitivity (1.0 pg/μL) and speed (~40 min) [104]. The assay has been validated in pilot POC detection of a food processing factory, supporting its potential applications in customs, quarantine units as well as meat import or export sectors [104]. Additionally, a CRISPR/Cas12a-driven surface-enhanced Raman scattering (SERS) biosensor has been successfully developed for goat milk authenticity detection with an ultra-low detection limit of 224 aM [105]. The CRISPR/Cas12-based strategies can also potentially be applied to the detection of gene-edited foods. However, CRISPR/Cas-based methods are generally qualitative and the interpretation of results can be subjective as each person may interpret color changes differently. qPCR is a well-known method for quantifying milk/meat product components, but it requires specialized instruments and skilled personnel. Strategies like the warm-start rapid digital CRISPR approach (WS-RADICA) [63] and microfluidics-enabled digital isothermal Cas13a assay (MEDICA) [152] may hold great promise as the next-generation nucleic acid quantification approaches alternatives to qPCR [63,152], since they had lower detection limits and greater inhibitor tolerance than a bulk isothermal amplification-combined CRISPR/Cas-based assay and had similar performance to RT-dPCR and RT-qPCR [63,72,152].

## 4. Conclusions and Future Prospects

Rapid detection of infectious diseases is highly required in diagnosis and infection prevention, not only in humans, but also in livestock. Meat/milk authenticity and composition should also be evaluated quickly, reliably, and cost-effectively from public health perspectives and from religious perspectives. The sex determination and control of early embryos also has immense impact on global livestock production. Nucleic acid detection methods combining isothermal amplification and CRISPR/Cas systems have emerged in recent years, with robustness, convenience, sensitivity, specificity, affordability, and potential adaptation for on-site detection. The strategies mainly employ the target-activated trans-cleavage activities of Cas12 and Cas13, which can efficiently cleave ssDNA or ssRNA sequences. However, these approaches still have space for further improvement to reduce the chance of contamination and cost, and to increase speed and sensitivity. For example, the combination of using suboptimal PAM and RAVI-CRISPR could be tested to achieve nucleic acid extraction-free one-tube visual colorimetric detection. Naked-eye colorimetry of RAVI-CRISPR as a readout method could be widely applied to reduce costs, instead of using lateral flow strips or fluorescence detectors. In addition, a number of CRISPR-Cas12a/Cas13a-based amplification-free platforms have been successfully applied for human virus diagnostics, but their potential in nucleic acid detection in livestock has not yet been explored. The multiplexed pathogen detection system CARMEN also has great potential to simultaneously detect all important livestock-associated viruses and to comprehensively identify their variant subtypes.

## Figures and Tables

**Table 1:** Summary of reported CRISPR/Cas-based diagnostic platforms and their detection modes.

Nuclease	Platform Name	Target	Amplification Method	Readout Method	Mechanism	Model Organism	Year	Refs.
Cas9	NASBACC	RNA	NASBA	Colorimetry	PAM identification and cleavage to trigger Toehead switch	Zika Virus	2016	[33]
	DNA-FISH	DNA	-	Fluorescence	dCas9/sgRNA complex serves as targeting material, SYBR Green I works as fluorescent probe	MRSA	2017	[34]
	PC REPORTER	DNA	PCR	Luminescence	dCas9 linked to the N-terminal and C-terminal halves of firefly luciferase are programmed with sgRNA complementary to the up- and down-stream fragments of target DNA sequence to induce luminescence after colocalization	*Mycobacterium tuberculosis*	2017	[35]
	CAS-EXPAR	DNA, RNA	EXPAR	Fluorescence	Cas9 generates nicks and NEase cycling generates ssDNA internal primers	*Listeria monocytogenes*	2018	[36]
	ctPCR	DNA	PCR	Electrophoresis/qPCR	Amplification of target DNA by PCR1 with a pair of universal primers, treatment of PCR1 products via a procedure of Cas9 cleavage, A tailing and T adaptor ligation, and amplification of the treated DNA by PCR2 using universal specific primers	HPV 16/18	2018	[37]
	CRISDA	DNA	SDA	Fluorescence	Cas9 forms notches at the boundary, target gene is amplified by external primers	SNPs	2018	[38]
	CARP	DNA	PCR	Electrophoresis/qPCR	Cleavage of target DNA with Cas9 targeted by a pair of sgRNAs, ligation of the cleaved DNA using DNA ligase, PCR amplification of the ligated DNA	HPV 16/18	2018	[39]
	ctPCR3.0	DNA	qPCR	qPCR	Amplification of Cas9/sgRNA-cleaved DNA sequences by qPCR	HPV 16/18	2018	[40]
	FLASH-NGS	DNA	PCR	NGS	The cDNA/gDNA is blocked by phosphatase processing and digested by Cas9 with a set of gRNA targeting the gene of interest. Ligation of sequencing adaptors, amplification, sequencing follows.	Antimicrobial resistance genes	2019	[41]
	CRISPR-Chip	DNA	-	Potentiometry	CRISPR-Chip biosensor utilizes the gene targeting ability of catalytically deactivated sgRNA-CRISPR/Cas9 and anchored to a transistor to produce a label-free test device. The output signal is monitored by a simple handheld reader.	SNPs	2019	[42]
Cas 12	DETECTR	DNA	RPA	Fluorescence	Combining activation of Cas12a ssDNase with isothermal amplification	HPV 16/18	2018	[31]
	HOLMES	DNA, RNA	PCR	Fluorescence	Cas12a/crRNA binds to target DNA, which trans-cleaves non-target ssDNA, illuminating fluorescent signal.	JEV	2018	[43]
	CDetection	DNA	RPA	Fluorescence	Combining optimized tuned gRNA enables distinguishing differences at single base level	HPV16/18	2019	[44]
	E-CRISPR	DNA, protein	Amplification free	Electrochemical	Cas12a converts target identification activity into detectable electrochemical signal via an interrogating electrode which is constructed from non-specific ssDNA	DNA: HPV16, PB19; Protein: TGFβ1	2019	[45]
	CRISPR-responsive hydrogel	DNA/RNA	RPA/RT-RPA	μPAD readout	Upon activation with input defined by gRNA, Cas12a cleaves DNA in the gel, translating biological information into material property changes	Ebola	2019	[46]
	HOLMESv2	DNA, RNA	LAMP	Fluorescence	Upgrade of HOLMES/ integration of LAMP and Cas12b trans-cleavage into a single step	JEV	2019	[47]
	CRISPR-Cas12a-NER	RNA	RT-RAA	Fluorescent signals by Naked eye under blue light	When a target nucleic acid is present in the detection system, the quenched green fluorescent molecule-labeled ssDNA reporter is cleaved by Cas12a, resulting in green fluorescence visible to the naked eye	SARS-CoV-2	2020	[48]
	CASdetec	RNA	RT-RAA	Fluorescence from fluorescence reader or under blue light	Integrating sample processing protocols and nucleic acid amplification approaches with CDetection	SARS-CoV-2	2020	[49]
	STOP	RNA	RT–LAMP	Fluorescence, lateral flow assay	Combining simplified viral RNA isolation with isothermal amplification and CRISPR mediated testing	SARS-CoV-2	2020	[50]
	Cas-gold	DNA	RPA	Gold nanoparticle-based LFS test	Integration of Cas12a-based assay and gold nanoparticle based LFS	ASFV	2020	[51]
	Poly (A)- AuNPs	DNA	RPA	Naked eye	AuNP-based bioprobes with freezing-based labeling approach	ASFV	2020	[52]
	Electrochemical DNA biosensing	DNA	Amplification free	Differential pulse voltammetry	Binding to target DNA activates Cas12a ssDNase activity; the low surface coverage and non-compact morphological structure of the immobilized hpDNA electrochemical reporters provide exploitable substrates for efficient cleavage of Cas12a, resulting in a high-sensitive electrochemical DNA biosensor	HPV16/18	2020	[53]
	PGMs-CRISPR	RNA	RT-RAA	Glucose meter readout	Samples are rapidly pretreated and amplified by RT-RAA; the viral signal is converted to glucose signal by integrating CRISPR/Cas12a system and a glucose production reaction, allowing quantitative readout by a personal glucose meter	SARS-CoV-2	2021	[54]
	OR-DETECTR	RNA	RT-RPA	Fluorescence, Lateral flow assay	Single-tube assay platform based on RT-RPA and DNA endonuclease targeted CRISPR trans-reporter technology	SARS-CoV-2	2021	[55]
	opvCRISPR	RNA	RT-LAMP	Fluorescent detection by naked eye under blue light	Integrating RT-LAMP, Cas12a cleavage in single reaction system	SARS-CoV-2	2021	[56]
	MEF biosensor	DNA	Amplification free	Fluorescence	Metal-enhanced fluorescence through the use of DNA-functionalized Au-nanoparticles, and embedded DNA/RNA hairpin director for ultra-sensitive nucleic acid detection	DNA	2021, 2022	[57,58]
	CRISPR-ENHANCE	RNA	Amplification free	Fluorescence, lateral flow assay	Significantly high sensitivity was achieved using engineered crRNAs and optimized conditions, enabling nucleic acid detection at femtomolar levels even without target pre-amplification	SARS-CoV-2	2022	[59]
	MOPCS	RNA	Amplification free	Surface plasmon resonance signal	Coupling optical sensing “surface plasmon resonance” with CRISPR “gene scissors” for high sensitivity and specificity	SARS-CoV-2	2022	[60]
	RAVI-CRISPR	DNA/RNA	LAMP/RT-LAMP	Naked-eye colorimetric detection	A field deployable detection platform based on ROX-labeled reporter, isothermal amplification and CRISPR/Cas12a system; a convolutional neural network algorithm developed for standardizing and automating the analytical colorimetric evaluation of images and implemented into MagicEye cell phone software	SARS-CoV-2, ASFV	2022	[61]
	sPAMC	DNA/RNA	RPA	Fluorescence	Cas12a’s reduced binding affinity to suboptimal PAM substrates is critical for its diminished cis-cleavage activity, thereby facilitating an equilibrium shift to isothermal amplification, resulting in stronger fluorescence	SARS-CoV-2, HCMV	2022	[62]
	WS-RADICA	DNA/RNA			Evaluation of two digital chips for DNA/RNA quantification	SARS-CoV-2, human adenovirus, herpes simplex virus	2022	[63]
Cas 14	Cas14-DETECTR	DNA	RPA	Fluorescence	Cas14 protein can cleave ssDNA in a targeted manner without restrictive sequence requirements. Non-specific cleavage of ssDNA molecules is triggered by targeted recognition of Cas14, which activity allows high-fidelity SNP genotyping		2018	[64]
Cas13	SHERLOCK	DNA/RNA	RPA	Fluorescence	crRNA/Cas13 targets ssRNA and splits fluorescent ssRNA probe	ZIKV, DENV, KPC, NDM-1	2017	[65]
	SHERLOCKv2	DNA/RNA	RPA	Lateral flow assay	Upgrade of SHERLOCK; high quantitation, high sensitivity	ZIKV, DENV	2018	[66]
	HUDSON + SHERLOCK	DNA/RNA	RPA	Fluorescence	Pairing HUDSON and SHERLOCK enables instrument-free detection of viruses directly from body fluids	ZIKV, DENV, WNV, YFV	2018	[67]
	CARMEN	DNA/RNA	PCR/RPA	Fluorescence	Over 4500 nucleic acids in one array by SHERLOCK methodology	HCV, HIV, ZIKV, DENV, influenza, SARS	2020	[68]
	SHINE	RNA	RPA	Smartphone (in-tube fluorescence readout or lateral flow strip)	Modified HUDSON quickly deactivates viruses in samples such as saliva and nasopharyngeal swabs in 10 min, and target RNA detection results are visualized by in-tube fluorescent readout and interpreted by a mobile app	SARS-CoV-2	2020	[69]
	Electrochemical CRISPR/CHDC system	RNA	-	Electrochemical readout	Dual signal enhancement strategy (CRISPR/Cas13a system plus catalytic hairpin DNA circuit) embedded in a re-usable electrochemical biosensor to rapidly and accurately detect target RNAs	NSCLC-related RNAs	2021	[70]
	OR-SHERLOCK	RNA	RPA	Fluorescence/Lateral flow assay	Single-tube assay platform based on RT-RPA and CRISPR/Cas12a	SARS-CoV-2	2021	[55]
	Multiple enhanced CRISPR-Cas13 assay	RNA	Amplification free	Fluorescence measurement by mobile phone camera with additional optics	Non-amplification CRISPR/Cas13a test for direct measurement from nasal swab RNA, readable with a cell phone microscope	SARS-CoV-2	2021	[71]
	Ultralocalized Cas13a Assay	RNA	Amplification free	Fluorescent microscopy (digital droplet readout)	Enclosing RNA-triggered Cas13a catalytic system in cell-like sized reactors by droplet microfluidics to simultaneously increase local concentrations of targets and reporters	SARS-CoV-2	2021	[72]
	gFETs	RNA	Amplification free	Fluorescence	By utilizing Cas13a’s transcleavage mechanism and ultra-sensitive Graphene field effect transistors	SARS-CoV-2, RSV	2022	[73]
	CRISPR-Cas13a/HRP assay	DNA/RNA	Amplification free	Naked-eye colorimetric detection	Coupling target induced Cas13 activity with subsequent release into solution of the enzymatic reporter HRP	SARS-CoV-2	2022	[74]

CRISPR, clustered regularly interspaced short palindromic repeats; NASBA, Nucleic acid sequence-based amplification; EXPAR, exponential amplification reaction; SDA, strand displacement amplification; (RT-)RPA, (reverse transcription) recombinase polymerase amplification; RCA, rolling circle amplification; (RT-)LAMP, (reverse transcription) loop-mediated isothermal amplification; (RT-)RAA, (reverse transcription) recombinase-aided amplification; qPCR, quantitative PCR; PAM, protospacer adjacent motif; LFS, lateral flow strip.

**Table 2:** Overview of applications of CRISPR-based nucleic acid detection in livestock.

Species	Detection Target	Assay Name	Target Region	Nucleic Acid Amplification	CRISPR Protein	Readout	LOD	Testing Time	One Tube vs. Two Tubes	Year	Refs.
Detection of pathogenic viruses											
Pig	ASFV	CRISPR/Cas9 eraser-based PAM-implanted PCR visual end-point detection	*p72*	PAM-implanted PCR	Cas9 eraser	LFA			One	2021	[75]
Pig	ASFV	RAVI-CRISPR	*p72*	LAMP	Cas12a	Naked-eye colorimetric readout	7 total copies	35 min	One	2020, 2022	[61,76,77]
Pig	ASFV	CRISPR/Cas13a-LFD	*p72*	RAA	Cas13a	LFS visual readout	10^1^ copies/µL	<1 h	One	2022	[78]
Pig	ASFV	multiplex-crRNA CRISPR/Cas12a system	*B646L*	Amplification-free	Cas12a	LightCycler 96	1 pM			2022	[79]
Pig	PRRSV	Highly sensitive CRISPR/Cas12a-Based Fluorescence detection	*nsp2*	RT-RPA	Cas12a	Fluorescent readout	1 copy	25 min	One	2021	[80]
Pig	PRRSV	enhanced Cas13a lateral flow detection	*M*	RPA	Cas13a	Lateral flow, fluorescence	172 copies/μL			2020	[25]
Pig	PEDV	RT-ERA-CRISPR/Cas12a detection	*ORF3*	RT-ERA	Cas12a	Visual detection under LED blue light	2 copies	30 min		2021	[81]
Pig	PEDV	RT-RAA- CRISPR/Cas12a assay	*S*	RT-RAA	Cas12a	Fluorescence, visual, UV light, or flow strip detection	100 copies	1.5 h		2022	[82]
Pig	PEDV, TGEV, PDCoV, SADS-CoV	RT-LAMP-CRISPR/Cas12a	*ORF3*, *N*, *N*, *N*	Multiplex RT-LAMP	Cas12a	Naked-eye colorimetric detection	1 copy	25 min		2022	[83]
Pig	PCV3	ERA-CRISPR/Cas12a assay		ERA	Cas12a	Under UV/LED-blue light	7 copies	<1 h		2021	[84]
Pig	PPV	ERA-CRISPR/Cas12a system	*VP2*	ERA	Cas12a	Lateral flow detection	3.75 × 10^2^ copies/μL			2022	[85]
Pig	JEV	RAVI-CRISPR	*C*	RT-LAMP	Cas12a	Naked-eye colorimetric readout	8.97 total copies	1 h	One	2022	[86]
Pig	EMCV	RAA-CRISPR/Cas13a assay		RAA	Cas13a	LFS	10^1^ copies/µL	1 h		2022	[87]
Cattle	LSDV	RPA-Cas12a-fluorescence assay	*orf068*	RPA	Cas12a	Fluorescent signal	100 TCID_50_/mL	15 min	Two	2022	[88]
Cattle	BVDV	LwCas13a-based detection system	reported BVDV sequence in 5′UTR conserved region	-	Cas13a	Fluorescence	10^3^ pM	-		2021	[89]
Cattle	CaPV	LAMP-CRISPR/Cpf1 fluorescence detection		LAMP	Cas12a	Fluorometer, lateral flow test	1.47 × 10^−3^ TCID_50_	50 min		2022	[90]
Detection of pathogenic bacteria and parasites											
Pig, Cattle, etc.	Toxoplasma gondii	RPA-CRISPR/Cas12a assay	*B1*	RPA	Cas12a	Fluorometer or LFS	3.3 copies/μL	-	One	2022	[91]
Pig, Cattle, etc.	Toxoplasma gondii	RAA-Cas12a assay	*RE*	RAA	Cas12a	Fluorescence detection	1 fM	~1 h		2021	[92]
Pig, Cattle, etc.	Toxoplasma gondii	RAA-Cas13a-LFD assay	*B1*	RAA	Cas13	LFD	1 × 10^−6^ ng/μL	<2 h		2022	[93]
Pig, Cattle, etc.	Cryptosporidium parvum IId-subtype-family	ReCTC-based diagnoses	*GP60*	RPA	Cas12a	LFS biosensor	single copy			2021	[94]
Pig, Cattle, etc.	Brucellosis	Dual- biosensors based on RPA-CRISPR/Cas12a		RPA	Cas12a	Fluorescent biosensor, electrochemical biosensor	2 copies			2022	[95]
Pig, Cattle, etc.	Escherichia coli, Streptococcus aureus	RPA-CRISPR/Cas12a	*rfbE*, *nuc*	RPA	Cas12a	Fluorescence	1 CFU/mL	<50 min	One	2020	[96]
Pig, Cattle, etc.	Escherichia coli	RAA-CRISPR/Cas12a	*wzy*	RAA	Cas12a	Fluorescence	5.4 × 10^2^ CFU/mL	30 min		2022	[97]
Pig, Cattle, etc.	Campylobacter jejuni	RAA-CRIPSR/Cas12a	*hipO*	RAA	Cas12a	Fluorescence	5 copies	15–30 min		2022	[98]
Pig, Cattle, etc.	Listeria monocytogenes	RPA-CRISPR/Cas12a		RPA	Cas12a	Fluorescence	10 CFU/mL			2021	[99]
Pig, Cattle, etc.	Yersinia enterocolitica	RPA-CRISPR/Cas12a	*ail*	RPA	Cas12a	Fluorescence	1.7 CFU/mL	<45 min		2022	[100]
Other applications											
Pig	Sex determination	RAVI-CRISPR	*SRY*, *ZFX*	LAMP	Cas12a	Fluorescence	2 copies	~45 min to 1 h		2022	[101]
Pig	Pig-derived component	CAPCOD		PCR	Cas12		0.1% (*w*/*w*)			2022	[102]
Pig	Pig-derived component	RPA-CRISPR/Cas12a assay		RPA	Cas12a	Visual identification	0.1–0.001% (*w*/*w*)	<30 min		2022	[103]
Pig, Chicken, Duck	Meat species	RAVI-CRISPR	porcine *NADH4*, chicken *ND2*, duck *D-loop*	LAMP	Cas12a	Naked-eye colorimetric detection	1.0 pg gDNA	40 min		2022	[104]
Cattle	Milk authenticity	CRISPR/Cas12a-Driven SERS Biosensor	*cytb*	LAMP	Cas12a	Spectrometer	224 aM			2022	[105]

CRISPR, clustered regularly interspaced short palindromic repeats; (RT-)LAMP, (reverse transcription) loop-mediated isothermal amplification; (RT-)RPA, (reverse transcription) recombinase polymerase amplification; (RT-)RAA, (reverse transcription) recombinase-aided amplification; (RT-)ERA, (reverse transcription) enzymatic recombinase amplification; PAM, protospacer adjacent motif; LFS, lateral flow strip; LFA, lateral flow nucleic acids assay.
